# Resolution of Treatment-Resistant Vertigo and Suspected Ménière’s Disease Following Knee Chest Upper Cervical Chiropractic Care: A Case Report

**DOI:** 10.7759/cureus.113493

**Published:** 2026-07-28

**Authors:** Shaan Rai

**Affiliations:** 1 Chiropractic, Vitality Chiropractic Centre, Singapore, SGP

**Keywords:** bournemouth questionnaire, cervicogenic dizziness, chiropractic singapore, dizziness, knee chest upper cervical technique, ménière's disease, tinnitus, upper cervical chiropractic, vertigo, vestibular migraine

## Abstract

Ménière's disease and related peripheral vestibular disorders are notoriously difficult to manage, and many patients report persistent symptoms despite pharmacological treatment. Cervicogenic mechanisms have been proposed as contributing factors in some presentations of chronic dizziness, and a small body of literature has explored upper cervical chiropractic care as a conservative intervention.

We report the case of a 44-year-old female office worker with a three-year history of episodic vertigo, imbalance, right-sided tinnitus, aural fullness, headache, and anxiety, carrying a working medical diagnosis of Ménière’s disease and showing limited response to medication. Examination revealed upper cervical postural asymmetry, restricted cervical range of motion, positive modified Romberg and balance testing, and thermographic findings consistent with upper cervical dysfunction. The patient underwent knee chest upper cervical (KCUC) chiropractic care, with outcomes tracked using the Bournemouth Questionnaire (BQ) and serial clinical reassessment. Over 36 visits, BQ score improved from 60/70 at baseline to 17 at discharge from the initial care plan, with a marked improvement in vertigo frequency, balance, tinnitus, sleep, and anxiety. Partway through care, the patient was diagnosed with breast cancer and underwent lumpectomy and radiotherapy; overall gains were largely sustained, though some objective findings showed mild regression coinciding with this diagnosis and its associated stress.

This case documents a temporal association between KCUC chiropractic care and symptom improvement in a patient with suspected Ménière’s disease, with vestibular migraine as an unexcluded differential. The findings are hypothesis-generating only. Controlled studies are needed to clarify causality.

## Introduction

Ménière’s disease is a chronic disorder of the inner ear characterized by recurrent episodes of spontaneous vertigo, fluctuating low- to medium-frequency sensorineural hearing loss, tinnitus, and aural fullness [1]. Diagnostic criteria established by the Bárány Society distinguish “definite” from “probable” Ménière’s disease on the basis of audiometric confirmation and the duration and pattern of vertigo attacks, with definite disease requiring audiometrically documented low- to mid-frequency sensorineural hearing loss in the affected ear on at least one occasion [1]. These criteria were subsequently endorsed by the 2020 American Academy of Otolaryngology - Head and Neck Surgery (AAO-HNS) Clinical Practice Guideline, which updated management recommendations while retaining the 2015 classification framework [2]. The condition can be profoundly disabling, with unpredictable attacks affecting occupational function, driving safety, and psychological well-being.

Psychological stress has long been recognized as both a consequence and a possible trigger of Ménière’s disease activity. A vicious cycle has been described in which the somatic symptoms of the disease provoke anxiety, which in turn appears to aggravate disease activity [3]. A large longitudinal study using daily symptom tracking found that unusual life events and elevated stress levels were associated with significantly increased odds of vertigo attacks and greater symptom severity, with measurable 24-hour lead and lag effects [4]. This relationship is relevant to the present case, in which a major intercurrent stressor occurred during the course of care.

Separately, a body of literature has explored the contribution of cervical spine dysfunction to chronic dizziness, sometimes termed cervicogenic dizziness (CGD): a disorder in which dizziness is associated with neck pain or dysfunction, attributed to disrupted proprioceptive input from the cervical spine interacting maladaptively with the vestibular and visual systems [5]. Some authors have gone further, proposing that a subset of cases diagnosed as Ménière’s disease may, in fact, represent a cervicogenic process, describing a conceptual overlap between “cervicogenic endolymphatic hydrops” and classical Ménière’s disease [6]. Case reports describing resolution of cervicogenic dizziness following conservative cervical spine management, including chiropractic care, have been published in patients with confirmed cervical pathology or instability and an exclusionary diagnostic workup [7,8]. A recent narrative review examined upper cervical segmental dysfunction, National Upper Cervical Chiropractic Association (NUCCA)-style corrective intervention, and dizziness outcomes, highlighting the limited but growing literature connecting upper cervical correction to vestibular symptom improvement, including in patients with a Ménière’s-type presentation [9].

The knee chest upper cervical (KCUC) technique is a specific-contact, high-velocity, low-amplitude (HVLA) upper cervical adjusting approach in which the patient is positioned prone in a knee-chest posture, with the head rotated to one side rather than facing directly downward, and a controlled, short-amplitude thrust is delivered to the atlas or axis. Listings (the specific vertebral level, contact point, and correction vector) are initially assessed using postural analysis, leg length comparison, manual muscle testing, balance testing, and thermographic scanning, and are subsequently confirmed by upper cervical radiographic analysis prior to adjustment. For atlas (C1) corrections, contact is made on the posterior arch with a predominantly vertical force vector; for axis (C2) corrections, contact may be made on the transverse process, spinous process, or posterior arch depending on the listing, with a force vector that is both vertical and directed inferior-to-superior. Listings are reassessed after adjustment using the same objective indicators, including thermographic scanning, postural analysis, and comparative leg length assessment. A 2025 narrative review of upper cervical chiropractic care and dizziness identified foundational case-level evidence for NUCCA-style intervention in vestibular presentations but noted the absence of high-quality studies and called for further case reports and observational research [9]. To our knowledge, no published case report has specifically described KCUC outcomes in a patient with a Ménière’s-type vestibular presentation.

We report a case of treatment-resistant vertigo and suspected Ménière’s disease, refractory to standard medical management, that showed substantial and largely sustained improvement during a course of KCUC chiropractic care despite the complicating onset of breast cancer during treatment. This case report was prepared in accordance with the CARE (CAse REport) guidelines [10]. Written informed consent was obtained from the patient for publication of this case report and any accompanying clinical data.

## Case presentation

Patient information

A 44-year-old female executive assistant presented for chiropractic consultation in March 2024 with a three-year history of episodic vertigo and associated symptoms.

History of presenting complaint

The patient’s symptoms began in March 2021 with episodes of spinning sensation occurring in the morning and lasting approximately one week. Initial evaluation by her general practitioner did not identify a clear cause, and breathing exercises provided partial relief. The condition relapsed in early 2023. By the time of presentation, the patient was experiencing vertiginous attacks approximately once per month, characterized by transient visual blanking (noted here as a reported associated feature, not a cardinal diagnostic criterion of Ménière’s disease, and one whose presence also raises vestibular migraine as a differential diagnosis), a vibrating sensation in the head, right-sided tinnitus, and imbalance. Symptoms had acutely worsened from January 5, 2024, with daily symptoms throughout that month.

Associated symptoms included insomnia (five to six hours of non-restorative sleep per night, with long-standing sleep disturbance and occasional sudden waking), anxiety, migraine-type headaches occurring several times per week (bilateral, occipital, tension-type, unresponsive to paracetamol), bilateral neck and right shoulder stiffness (worse with heavy lifting or prolonged desk work, improved with stretching, associated with intermittent right arm numbness exacerbated by phone use), a sensation of leg weakness, early satiety with indigestion, bloating and nausea, intermittent shortness of breath during symptomatic episodes, and dysmenorrhoea. The right ear felt blocked, pressured, and full. The patient reported that her symptoms fluctuated between better and worse days and described a significant impact on her ability to concentrate, maintain balance, sleep, work, socialize, and travel, with associated low mood.

The patient’s general practitioner had raised a working diagnosis of Ménière’s disease, and she had also been evaluated by an otolaryngologist. This diagnosis was established by the patient’s medical team; independent audiometric confirmation of endolymphatic hydrops was not available to the authors for this report. Prior management consisted of medication trials with limited response. At presentation, she was taking betahistine (dose not documented in the clinical records available to the authors), paracetamol, and an unspecified sleep aid. Following radiographic workup, she was additionally prescribed hydroxyzine, a vasodilator/nootropic agent, and prochlorperazine by her treating physicians.

Past medical, surgical, family, and social history

The patient reported a known dairy intolerance and no relevant prior surgery. Family history was notable for paternal renal failure. She denied tobacco, alcohol, or substance use. Her lifestyle was sedentary, with a desk-based occupation (9 a.m. to 5 p.m. screen-based work) and only light stretching as regular physical activity. She had received chiropractic care on one occasion several years prior for postural complaints, without follow-up.

Physical examination

Postural assessment revealed translation of the head to the left and a low right shoulder. Cervical range of motion was restricted in extension, right lateral flexion, and left rotation, with pain reproduced on extension and right lateral flexion.

Balance was assessed using a modified cervical Romberg protocol. The standard Romberg test was first performed as a baseline (patient standing with feet together, arms at sides, eyes closed). The test was then repeated with the head held sequentially in six cervical positions: rotation to the left, left rotation with flexion, left rotation with extension, rotation to the right, right rotation with flexion, and right rotation with extension. Each position was assessed first with eyes open and then with eyes closed. The patient stood near a wall for safety, and the examiner remained in close attendance throughout. Feet were held together throughout; where significant balance impairment is present, a wider stance may be used for safety, though this was not required in this case. The purpose of this protocol is to determine whether cervical head position, rather than vestibular or central factors alone, contributes to balance impairment: worsening balance specific to particular cervical positions implicates the cervical proprioceptive system as a contributing factor. Positive findings may be subjective (reported dizziness, nausea, or sense of imbalance) or objective (observable sway, grabbing for support, or loss of balance). In this patient, balance was notably worse with the head rotated right, and further worsened with the addition of cervical flexion or extension in the right-rotated position, with no equivalent deficit in left-sided positions.

Cervical distraction testing was negative; cervical compression testing reproduced both imbalance and neck pain. Upper cervical manual muscle testing demonstrated weakness in the upper limb specifically when the head was held in combined right rotation and extension, with no weakness elicited in other head positions. Static bilateral weight-bearing assessment showed a 3.4 kg side-to-side discrepancy. Blood pressure was 133/84 mmHg with a pulse of 67 beats per minute. Prone leg length assessment showed a functionally short right leg that normalized with right cervical rotation, suggesting a cervical rather than structural origin. Paraspinal infrared thermography was performed using a dual-sensor scanning instrument that records bilateral skin temperature along the cervical paraspinal musculature and derives a delta-T (left-minus-right) differential trace. It is noted that paraspinal thermography is used within the KCUC clinical framework as an adjunct assessment tool to guide listing determination and monitor correction stability; its use as a validated diagnostic or outcome measure in vestibular disorders is not independently established, and findings should be interpreted accordingly. At initial consultation, repeated scans showed a discernible left-right temperature differential pattern along the upper cervical region, consistent with asymmetric paraspinal thermal activity and supportive of an upper cervical neurological component to the presentation within the clinical context of KCUC practice.

Oculomotor assessment showed normal smooth pursuit and divergence, with no nystagmus. Convergence testing provoked dizziness and discomfort. Saccadic eye movements were difficult for the patient to perform, though no flickering or dysmetria was noted.

Diagnostic imaging

**Figure 1:**
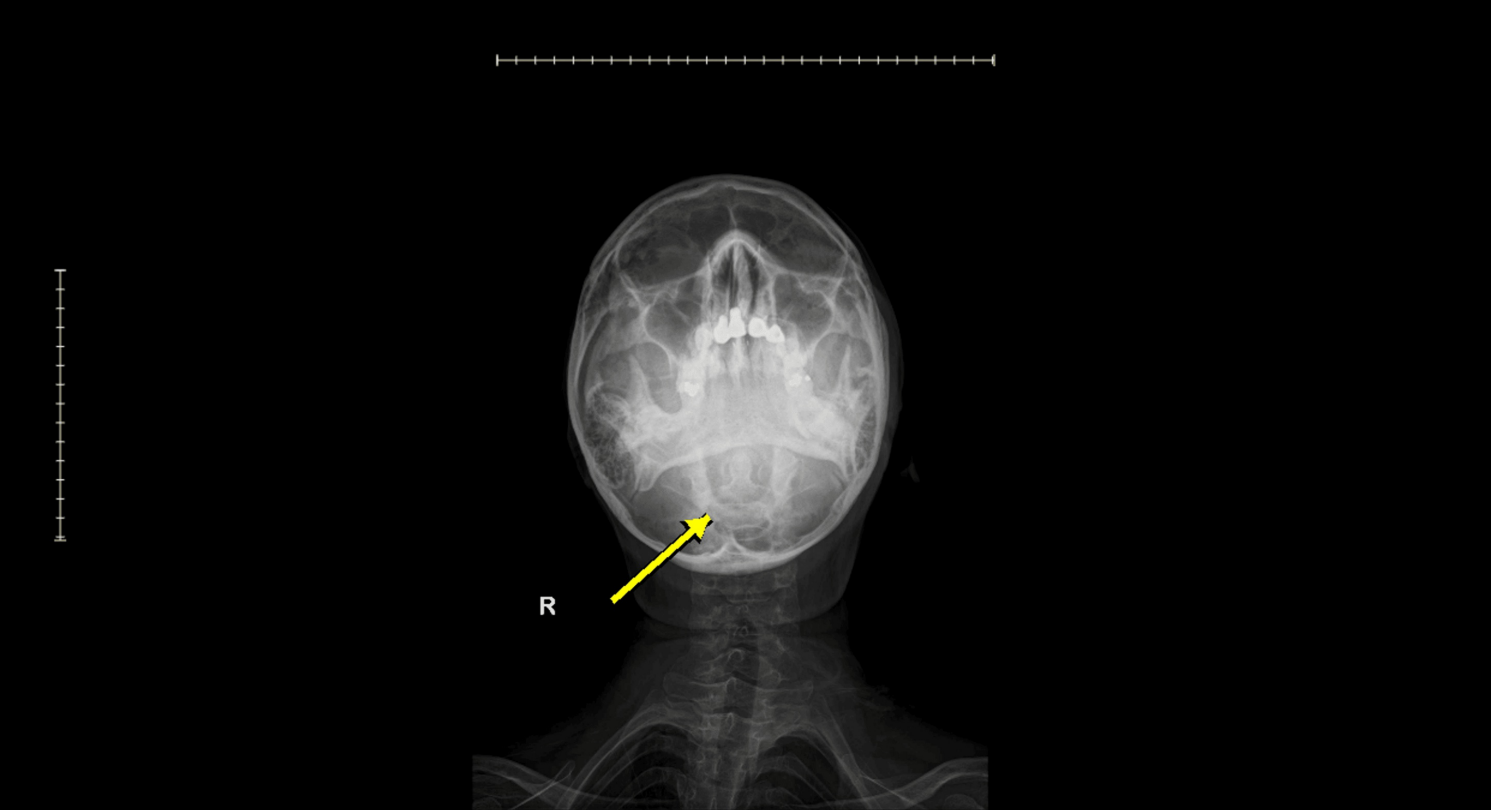
Base posterior (vertex) radiograph. Arrow indicates the upper cervical joint region.

**Figure 2:**
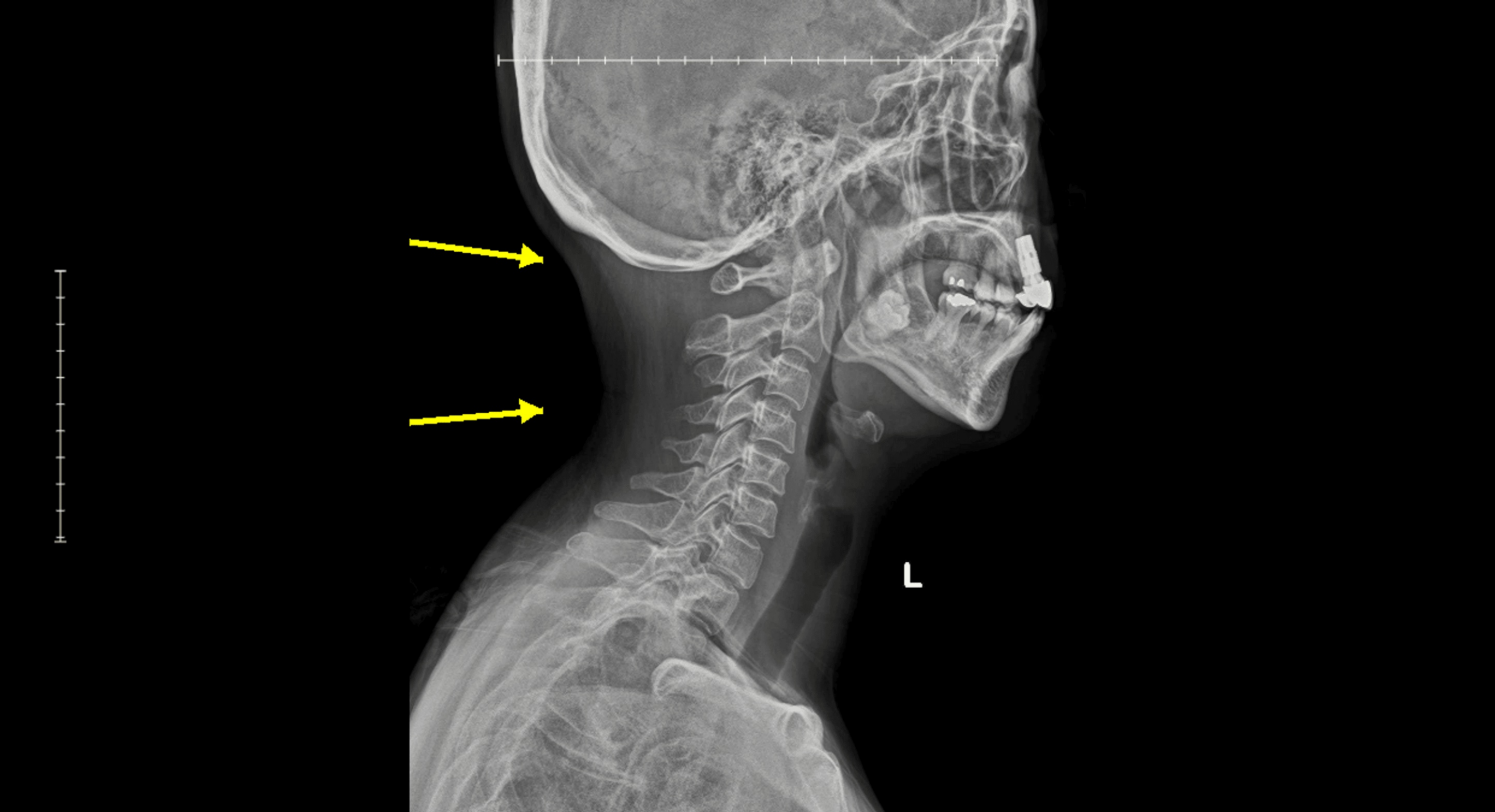
Lateral cervical radiograph demonstrating loss of normal cervical lordosis and straightening of the cervical spine. Arrows indicate the cervical spine, highlighting the absence of normal lordotic curve.

**Figure 3:**
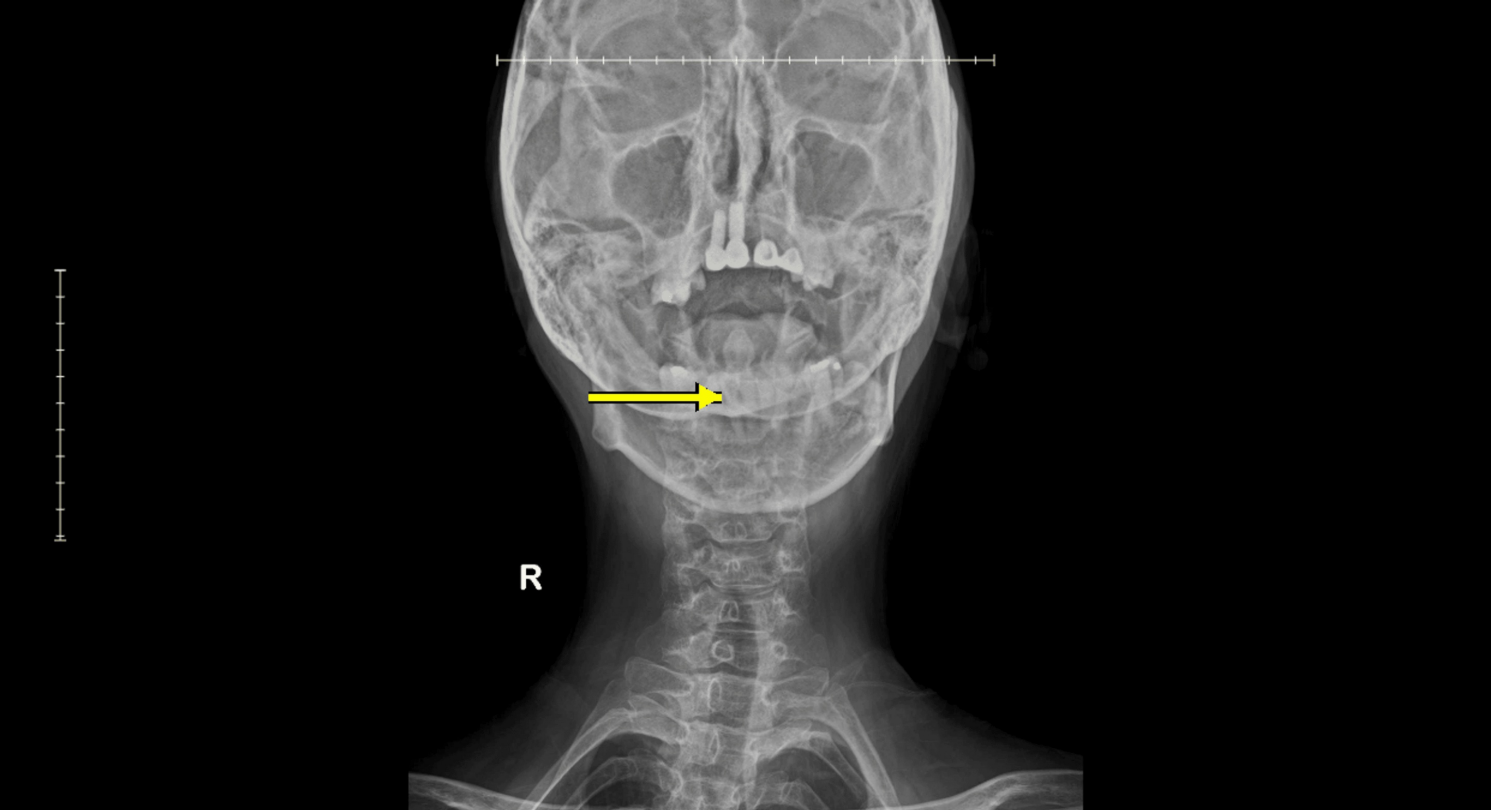
Anteroposterior open-mouth radiograph in neutral position. Arrow indicates the upper cervical region.

**Figure 4:**
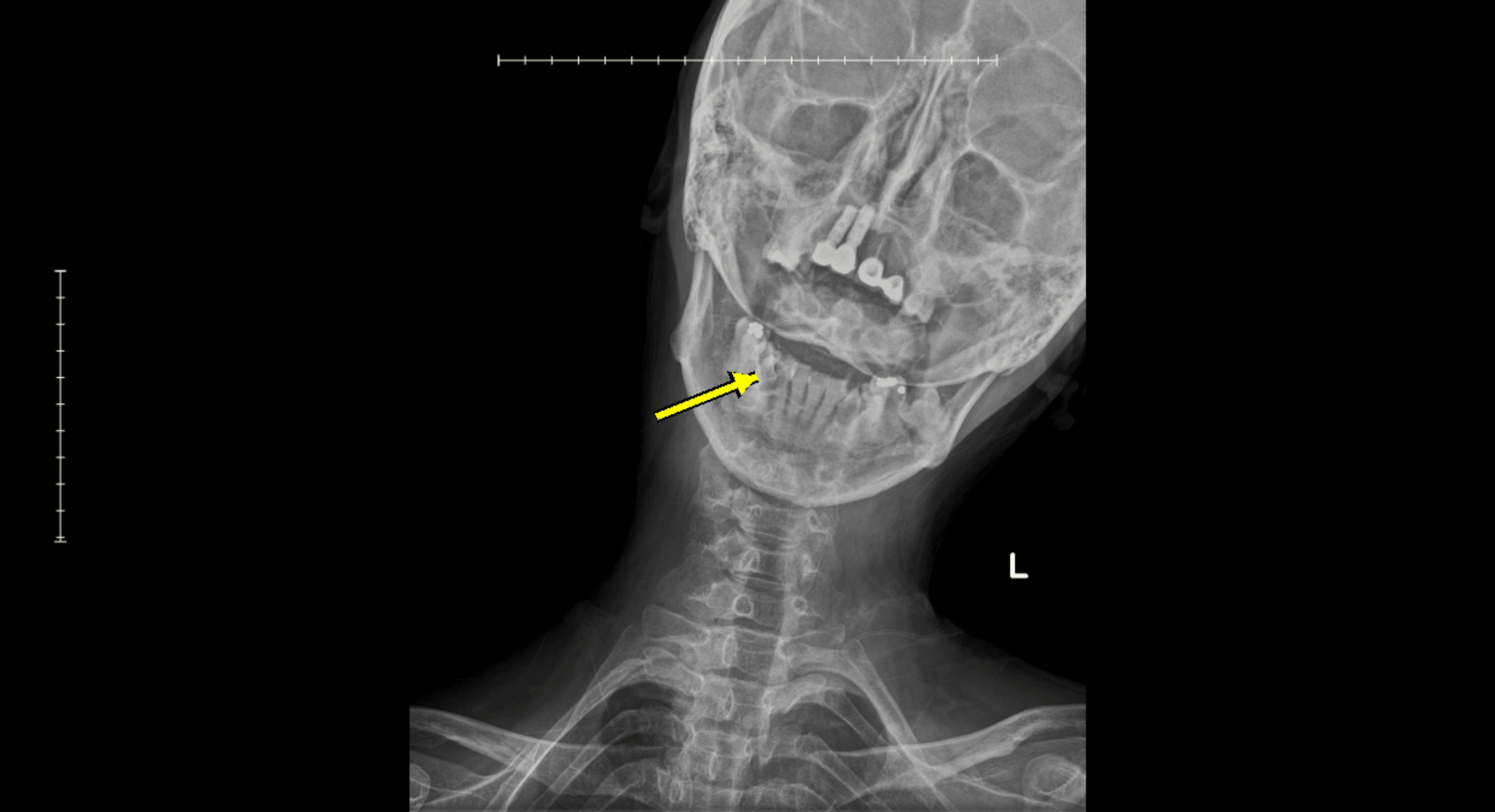
Anteroposterior open-mouth radiograph in left lateral flexion. Arrow indicates the upper cervical region.

**Figure 5:**
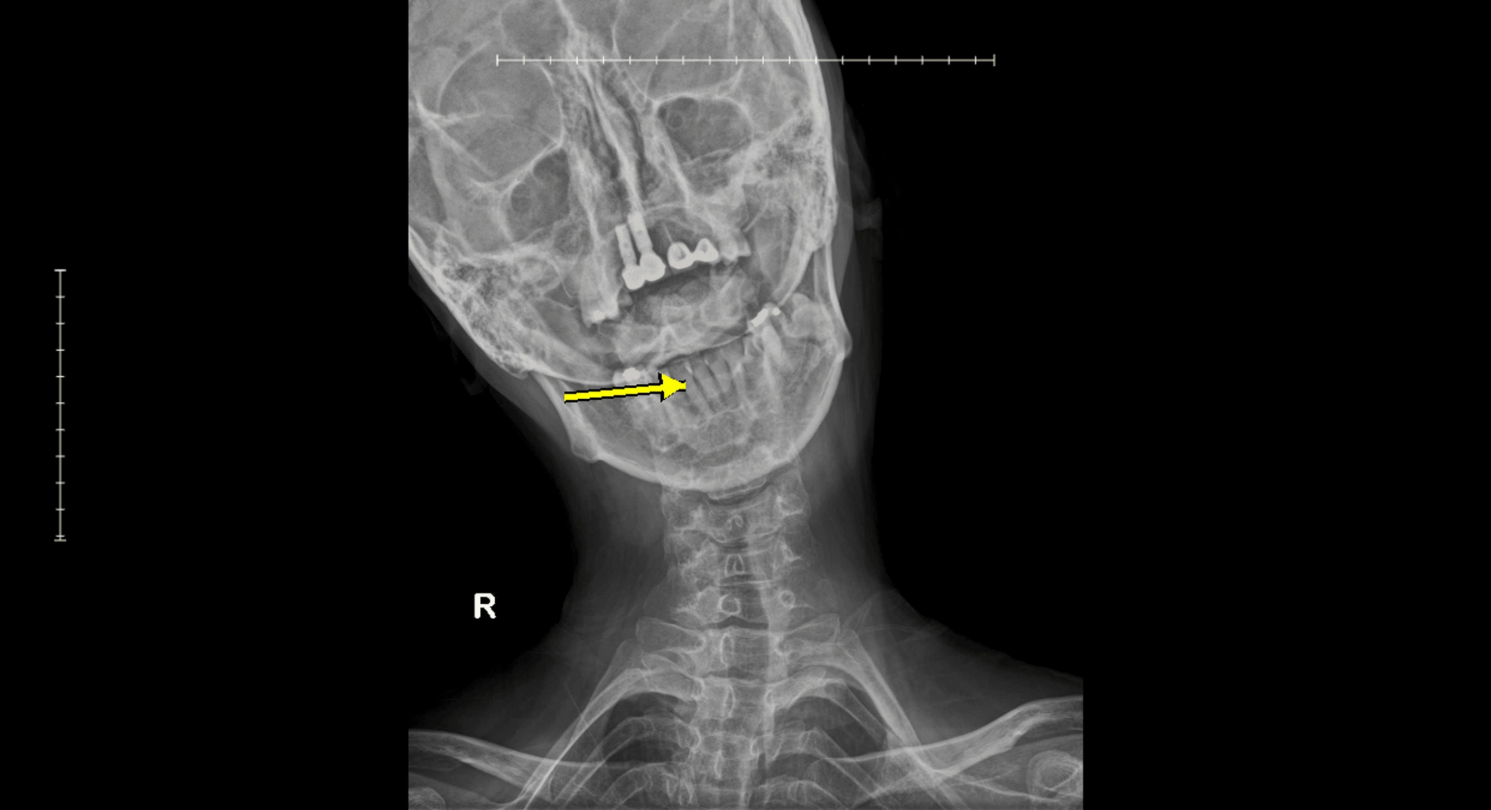
Anteroposterior open-mouth radiograph in right lateral flexion. Arrow indicates the upper cervical region.

Upper cervical radiographs were obtained on March 16, 2024, and included five views: lateral cervical, base posterior (vertex), anteroposterior open-mouth in neutral, and anteroposterior open-mouth in left and right lateral flexion (Figures 1-5). Radiologist interpretation reported loss of normal cervical lordosis with straightening of the cervical spine, and mild-to-moderate disc space narrowing with mild osteophytosis on a background of cervical spondylosis, without loss of vertebral body height. These degenerative findings are common in the general population at this patient’s age and are not in themselves specific to her vestibular presentation; they are reported as part of the standard upper cervical radiographic workup and listing analysis rather than as a proposed structural cause of her vertigo. The patient had been assessed by both a general practitioner and an otolaryngologist prior to chiropractic presentation; neither clinician had requested MRI or CT imaging, suggesting that central causes of vertigo - including posterior fossa lesions, acoustic neuroma, and demyelinating disease - had been clinically considered and not deemed to require advanced neuroimaging at that time. No magnetic resonance or computed tomography imaging was therefore available to the authors, and this is acknowledged as a limitation of the available clinical documentation.

Timeline

Table 1 presents a chronological summary of the key clinical events in this case.

**Table 1:** A chronological summary of the key clinical events in this case. BQ: Bournemouth Questionnaire; KCUC: knee chest upper cervical; TVP: transverse process.

Date	Event	Clinical Notes
March 2021	Symptom onset	Episodic morning vertigo, duration ~1 week; GP consulted, no clear cause identified
Early 2023	Symptom relapse	Recurrence of vertigo and associated symptoms
January 5, 2024	Acute deterioration	Daily vertigo, tinnitus, imbalance; worsened through January 2024
March 16, 2024	Initial chiropractic consultation (Visit 1)	Full history, examination, upper cervical radiographs obtained; BQ score 60/70; C2 left TVP listing identified
March 2024	Care commenced	KCUC adjustments initiated at C2 left transverse process; twice-weekly frequency
April 2024	Patient informs ENT	Patient reports to otolaryngologist that chiropractic care is providing benefit
May 18, 2024	1st reassessment (Visit 12)	Marked subjective improvement in vertigo, balance, anxiety; posture improved; modified Romberg improved; listing revised to C1 right posterior arch
June 14, 2024	2nd reassessment (Visit 24)	Patient reports near-full function, no triggers; treadmill exercise resumed; BQ improving; frequency reduced to once weekly
July 8, 2024	Breast cancer biopsy	Biopsy performed; result communicated 19 July 2024 - positive for breast cancer
August 14, 2024	Lumpectomy	Surgical intervention; patient attended chiropractic care the day prior
August 22, 2024	Care resumed post-surgery	Patient reports good recovery; chiropractic care continued alongside oncological management
September 20, 2024	3rd reassessment / discharge (Visit 36)	BQ score 17/70 (baseline 60/70); posture normalised; mild Romberg regression with left rotation; scale discrepancy 7 kg; radiotherapy commencing following week
September–October 2024	Radiotherapy course	20 sessions; maintenance chiropractic care continued throughout
~June 2025	Follow-up contact	Patient reports no recurrence of vertigo since discharge; mild shoulder tension and headache on starting new job, both self-resolving; breast cancer in remission

Clinical impression

Based on history, examination, and imaging findings, the chiropractic working diagnosis was upper cervical dysfunction with associated tension-type headache, in a patient carrying a separate medical diagnosis of Ménière’s disease. The upper cervical examination findings - including position-dependent imbalance and muscle weakness specific to right rotation and extension, asymmetric thermographic findings, and a functionally short leg that corrected with cervical rotation - were judged consistent with a cervical contribution to the patient’s vestibular and balance symptoms.

Treatment

The patient underwent care using the KCUC technique, as described in the Introduction. Repeat radiographs were not obtained during the course of care, as per standard practice, since no intervening physical trauma occurred that would have warranted re-imaging.

Care began with a C2 listing. Radiographic analysis indicated contact on the left transverse process of C2, with a correction vector directed vertically and inferior-to-superior. Early thermographic reassessments showed improvement following adjustment, but findings did not consistently hold between visits, requiring more frequent intervention. The listing was subsequently revised to C1 on the right following further radiographic analysis; for C1 corrections, contact is made on the posterior arch with a predominantly vertical force vector. This C1 listing held more durably and was maintained for the remainder of the initial care plan.

Following each adjustment, the patient was assisted to a dedicated recovery room and observed for 15-30 minutes before a post-adjustment thermographic scan was performed to assess whether the correction was holding. The standard supine bench position was not tolerated due to the patient’s vestibular symptoms, and an inclined recliner chair was used throughout the recovery period in place of the supine position.

If the initial thermographic scan does not indicate adjustment, the patient rests in the recovery room for approximately 10 minutes to reduce movement-related variables before a confirmatory rescan; this two-step scanning protocol helps distinguish a true negative finding from a transient artefact.

Care plan

Visit frequency began at twice weekly and was reduced according to reassessment findings, including thermographic stability (reduced need for adjustment, indicating the correction was “holding”), objective examination findings, and subjective symptom improvement. Frequency was reduced from twice weekly to once weekly following the second reassessment (24th visit). The patient completed a total of 36 visits over approximately four to six months.

Outcome measures

**Figure 6:**
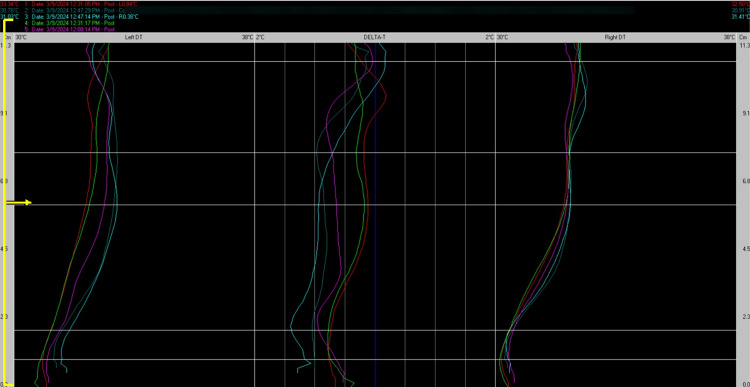
Paraspinal infrared thermography at initial consultation (March 9, 2024). Bracket indicates the full scan area assessed for deviation from delta, total width difference in paraspinal temperature, and baseline establishment. Practitioner comment text has been redacted to protect confidential patient information.

**Figure 7:**
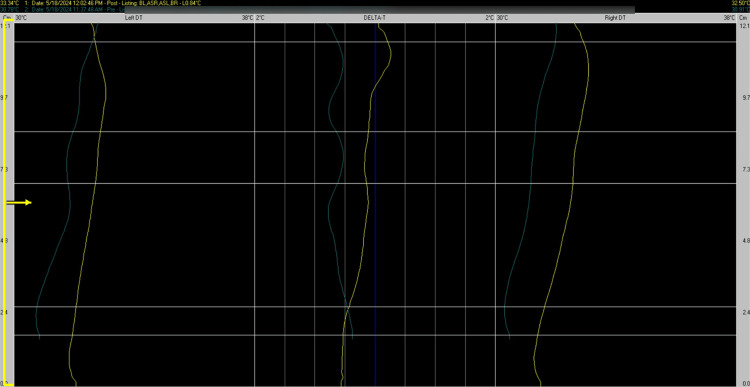
Paraspinal infrared thermography at the first reassessment (12th visit, May 18, 2024), pre- and post-adjustment. Bracket indicates the full scan area assessed for deviation from delta, total width difference in paraspinal temperature, and difference from baseline scans. Practitioner comment text has been redacted to protect confidential patient information.

**Figure 8:**
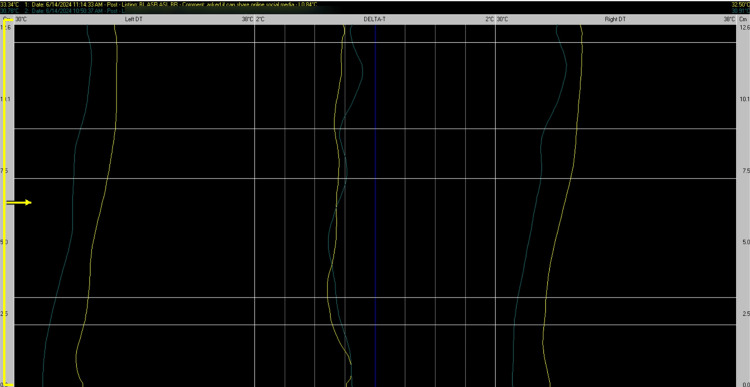
Paraspinal infrared thermography at the second reassessment (24th visit, June 14, 2024). Bracket indicates the full scan area assessed for deviation from delta, total width difference in paraspinal temperature, and difference from baseline scans. Practitioner comment text has been redacted to protect confidential patient information.

**Figure 9:**
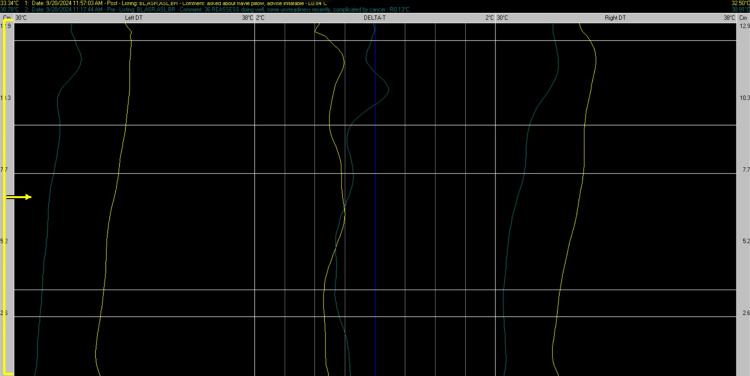
Paraspinal infrared thermography at the third reassessment (36th visit, September 20, 2024). Bracket indicates the full scan area assessed for deviation from delta, total width difference in paraspinal temperature, and difference from baseline scans. Practitioner comment text has been redacted to protect confidential patient information.

The Bournemouth Questionnaire (BQ), a validated seven-item patient-reported outcome measure assessing pain, disability, and cognitive/affective dimensions, was used to track progress [11]. Baseline BQ score at the initial consultation was 60/70. At discharge from the initial care plan (36th visit), BQ score had improved to 17/70. Serial paraspinal thermographic scans obtained at consultation and at each reassessment (Figures 6-9) were used alongside the BQ and clinical examination to monitor the cervical neurological component of the presentation over the course of care.

Clinical course

First Reassessment (12th visit, May 18, 2024)

The patient reported a marked reduction in vertigo, dizziness, giddiness, anxiety, and numbness, with improved energy, balance, and strength. She described improved ease with work, exercise, and concentration, and described her improvement as “huge.” She was walking further with greater ease, and her neck and shoulders felt softer, though intermittent tinnitus persisted. Objectively, posture had improved (shoulders level, though mild left head translation persisted), balance on modified Romberg testing was improved, and the weight-bearing scale discrepancy remained at 3.4 kg. Arm strength was normal. Cervical range of motion improved, with only cervical extension remaining restricted and left rotation mildly uncomfortable. Leg length was balanced.

Second Reassessment (24th visit, June 14, 2024)

The patient reported feeling “great,” with no symptom triggers identified, improved functional capacity, ability to exercise on a treadmill, improved sleep and appetite, and a sense of greater control over her life with improved mood. Mild residual neck and shoulder soreness persisted, but dizziness was described as vastly improved. Objectively, posture continued to improve (only slight left head translation, shoulders level), balance on modified Romberg was further improved, the weight-bearing scale discrepancy was 4 kg, and arm strength remained normal. Mild discomfort was noted on eye convergence testing. Leg length remained balanced. Cervical range of motion showed only left lateral flexion restricted and right rotation mildly uncomfortable.

Intercurrent Illness

During this period of care, the patient was diagnosed with breast cancer. She underwent biopsy on July 8, 2024, with a positive result communicated on July 19, 2024. Full histopathological classification and staging details were managed by the patient’s oncological team and were not available to the authors of this report. A course of 20 radiotherapy sessions and a lumpectomy were planned, with surgery performed on August 14, 2024. The patient attended chiropractic care the day before her surgery and resumed care on August 22, 2024, reporting a good recovery.

Third Reassessment (36th visit, September 20, 2024)

The patient reported feeling good overall and described the preceding period as “a big year for her health,” noting she was due to begin radiotherapy the following week and had experienced some mild dizziness at night in the preceding days. Objectively, posture showed no head tilt with level shoulders. Mild imbalance was noted on modified Romberg testing with left cervical rotation. The weight-bearing scale discrepancy had increased to 7 kg. Arm strength remained normal, cervical compression testing was now negative, and prone leg length was balanced. Eye movements were normal. Cervical range of motion showed only extension as mildly uncomfortable. BQ score at this visit was 17/70 (baseline 60/70).

Patient-reported experience

The patient described substantial improvement in her tinnitus, headaches, and nausea over the course of care, in her own words noting that the ringing in her ear, headaches, and nausea had all but resolved.

Adverse events

No adverse reactions or side effects related to chiropractic care were reported at any point during the course of treatment.

Discharge and follow-up

The patient continued under maintenance chiropractic care alongside her oncological treatment, with visit frequency progressively reduced to support recovery from radiotherapy and to help maintain her clinical gains. She continued to be followed by her otolaryngologist, oncologist, and radiation oncologist throughout this period, with a gradual reduction in her vestibular and anxiolytic medications; in April 2024, she informed her otolaryngologist that chiropractic care was providing benefit. At the time of writing, the patient remains in remission from breast cancer and continues with reduced-frequency maintenance chiropractic visits.

At approximately nine months post-discharge from the initial care plan, the patient reported that she had remained free of vertigo symptoms. She noted the onset of mild shoulder tension and a headache coinciding with starting a new job, both of which resolved quickly without requiring any change to her care schedule. No recurrence of vertigo, tinnitus, or imbalance was reported at this follow-up contact.

Patient perspective

The patient began seeking chiropractic care in March 2024 after three years of symptoms with limited relief from medical management. In a written progress report completed at the 24th visit (June 14, 2024, approximately three months into care), she described her condition at that point as follows: she was “able to function almost 100%,” with improved sleep and appetite, no identifiable symptom triggers, and the ability to exercise on a treadmill - activities she had not been managing prior to care. She noted that her mood had become “more positive and calm,” and reflected that “compared to the first visit, condition has improved tremendously - happy and glad I’ve more control of my life.” She also made lifestyle adjustments during this period, including regular exercise, reducing caffeine intake by switching to decaffeinated coffee, and eliminating alcohol, which she felt contributed to her overall improvement.

In a publicly available review written at the same timepoint, the patient described her experience in her own words: she had “yet to experience episodes of vertigo,” no longer felt “off balance and sway while walking,” and no longer felt “nauseous all the time.” She described feeling calmer and less anxious, and noted that her insomnia had improved. She reflected that she was “glad that I made the right decision and took the leap of faith to take up the treatment plan.”

## Discussion

This case describes substantial and largely sustained improvement in treatment-resistant vertigo, imbalance, tinnitus, and associated symptoms in a patient with a working medical diagnosis of Ménière’s disease, over a course of KCUC chiropractic care. The patient’s BQ score fell from 60/70 to 17/70, a magnitude of change well in excess of what would be expected from natural fluctuation alone, and was accompanied by convergent objective findings (postural correction, improved cervical range of motion, improved balance testing, resolution of cervical compression sensitivity) and a clear patient-reported improvement in quality of life.

The patient’s examination findings - position-dependent imbalance, weakness specific to combined right cervical rotation and extension, a functionally short leg that normalized with cervical rotation, and asymmetric thermographic findings localized to the upper cervical spine - are consistent with a cervicogenic contribution to her vestibular symptoms. We emphasize that this interpretation rests on these functional and neurological findings rather than on the non-specific degenerative radiographic changes noted on imaging, which are common at this patient’s age and were not treated as evidence of a structural cause. It must also be acknowledged that these clinical findings are not pathognomonic for cervicogenic dizziness and cannot independently establish the source of vestibular symptoms; they are consistent with, but do not confirm, a cervicogenic contribution. This aligns with a growing literature describing cervicogenic dizziness as a disorder in which disrupted cervical proprioceptive input interacts maladaptively with vestibular and visual processing, producing postural instability and dizziness in the absence of, or alongside, confirmed peripheral vestibular pathology [5]. Some authors have proposed a closer conceptual relationship still, suggesting that a meaningful subset of patients labeled with Ménière’s disease may have a primarily cervicogenic process, termed “cervicogenic endolymphatic hydrops” [6]. While this patient’s medical workup and diagnosis of Ménière’s disease were made independently by her treating physicians and are outside the scope of chiropractic diagnostic authority, her response to a purely cervical intervention is noteworthy in this context, and adds to a small but growing case-based literature exploring upper cervical chiropractic care in dizziness, including a recent narrative review specifically addressing upper cervical correction and Ménière’s-type presentations [9]. It should be noted that vestibular migraine represents a plausible alternative diagnosis that cannot be excluded without formal audiometric and vestibulometric testing; the phenotypic overlap between vestibular migraine and Ménière’s disease is well documented, and the presence of visual disturbance during vertiginous episodes in this patient further raises this differential. This diagnostic uncertainty is acknowledged as a limitation of the present report.

A particularly instructive feature of this case is the patient’s intercurrent diagnosis of breast cancer partway through her course of care. Despite this major physiological and psychological stressor - including biopsy, a positive cancer diagnosis, lumpectomy, and the anticipation of radiotherapy - the patient’s overall BQ score and most clinical markers continued to improve through to discharge. However, several objective findings showed mild regression at the third reassessment relative to the second: the weight-bearing scale asymmetry increased from 4 to 7 kg, and mild imbalance on modified Romberg testing reappeared with cervical rotation, alongside the patient’s report of recurrent mild dizziness in the days preceding that visit. A substantial, separate literature links psychological and physiological stress to Ménière’s disease activity independent of any cervical mechanism: a large longitudinal study found that unusual life events and elevated stress were associated with significantly increased odds of vertigo attacks and more severe symptoms, with measurable effects in the 24 hours before and after a stress exposure [4], and reviews of psychological factors in Ménière’s disease describe a bidirectional, self-reinforcing relationship between vestibular symptoms and anxiety [3]. We interpret the mild regression observed here as most consistent with this established stress-vertigo pathway operating alongside, rather than through, the cervical findings. The partial, modest nature of the regression - rather than a full relapse - may reflect the durability of the structural and neurological gains achieved earlier in care, even under significant biopsychosocial stress. To be clear, this proposed mechanism is explicitly hypothetical and not directly demonstrated in this single case. We do not suggest that chiropractic care addressed the cancer diagnosis or its psychological sequelae directly, nor that the cervical spine mediated the patient’s stress response to her diagnosis; rather, we hypothesise that KCUC care may have stabilized the upper cervical musculoskeletal and neurological substrate sufficiently to increase the resilience of the vestibular system to an independent, external stressor - a distinction important for the accurate interpretation of this case and one that requires controlled investigation before any causal inference can be drawn.

This case adds to a limited literature describing chiropractic management of dizziness and suspected Ménière’s disease, and is, to the authors’ knowledge, among a few published cases in which the influence of a major intercurrent medical diagnosis on the trajectory of upper cervical chiropractic care has been documented in detail. The clinical lesson for practitioners managing similar patients is twofold: first, that careful, structured reassessment, combining patient-reported outcome measures such as the BQ with objective clinical and instrumentation-based findings, can help distinguish a genuine treatment plateau or regression from the expected, transient effect of an external stressor; and second, that clinicians should anticipate and openly discuss with patients how major life stressors may temporarily affect symptom trajectory during care, to support realistic expectations and continued engagement with treatment.

Limitations

This is a single case report, and as such, no causal relationship between KCUC chiropractic care and the patient’s symptom improvement can be established. The BQ, while validated and responsive to change in musculoskeletal pain populations, was not developed or validated specifically for vestibular or Ménière’s disease symptoms, and was used here as a general pain/disability/affective outcome measure rather than a vestibular-specific instrument. Future similar cases should consider including a validated vestibular-specific measure such as the Dizziness Handicap Inventory (DHI), which would allow more granular tracking of functional, physical, and emotional dizziness-related disability across the care episode. The patient continued to receive concurrent medical care, including a gradual reduction in medication, oncological treatment, and follow-up with an otolaryngologist throughout the period described, and the relative contribution of these concurrent factors to her improvement cannot be fully disentangled from that of chiropractic care. The diagnosis of Ménière’s disease was made by the patient’s treating physicians and was not independently confirmed by audiometric or vestibular testing within this report. Advanced neuroimaging (MRI or CT) was not available to the authors; while the patient’s treating otolaryngologist did not refer for such imaging, its absence means that central causes of vertigo cannot be formally excluded within this report. As with all single case reports, these findings cannot be generalized to other patients.

## Conclusions

This case describes substantial and largely sustained improvement in vertigo, imbalance, tinnitus, headache, and anxiety in a patient with treatment-resistant, medically diagnosed Ménière’s disease, over a course of KCUC chiropractic care, accompanied by convergent objective and patient-reported outcome data. The case also illustrates the potential influence of a major intercurrent medical and psychological stressor on the trajectory of recovery. While encouraging, these findings represent a temporal association between the commencement of KCUC care and symptom improvement and should not be interpreted as evidence of treatment efficacy; natural disease fluctuation, concurrent medical management, lifestyle modifications, and placebo effects may all have contributed to the observed changes. Controlled studies are needed to clarify whether upper cervical chiropractic care has a genuine causal role in the management of cervicogenic or Ménière’s-type vestibular symptoms, and to identify which patients are most likely to benefit.
